# Omicron variant of SARS‐CoV‐2, an epidemiologic assessment of pediatric oncology patients in the Bronx

**DOI:** 10.1002/cnr2.1724

**Published:** 2022-10-05

**Authors:** Rachel Offenbacher, Joshua Gorlick, Daniel A. Weiser

**Affiliations:** ^1^ Department of Pediatrics, Albert Einstein College of Medicine and Division of Pediatric Hematology, Oncology and Cellular Therapy Children's Hospital at Montefiore Bronx New York USA; ^2^ Department of Pediatrics Albert Einstein College of Medicine, Children's Hospital at Montefiore Bronx New York United States

**Keywords:** bacteremia, neutropenia, omicron, Sars‐CoV2

## Abstract

**Background:**

Children receiving cytotoxic therapy for cancer have increased risk of infection due to drug‐induced neutropenia and are therefore treated empirically for bacteremia when febrile or ill‐appearing. However, viral infections, which are not frequently life‐threatening, are the most common etiology of febrile episodes and there has been increased effort to differentiate patients who may have a higher risk for adverse outcomes.

**Case:**

We performed a retrospective chart review of pediatric oncology patients diagnosed with COVID‐19 between December 20, 2021 and February 22, 2022 during the Omicron (B.1.1.529) surge at The Children's Hospital at Montefiore, a tertiary care center in the Bronx.

**Conclusion:**

We found that no patients in our cohort developed respiratory distress, bacteremia, or serious illness after COVID‐19 infection during the Omicron surge. Future studies will aid in understanding the relationship between community‐acquired infections and bacteremia, and this knowledge can then be applied to develop optimal infection prevention clinical care guidelines.

## 1 BACKGROUND

When children with cancer have a fever in the setting of chemotherapy‐induced neutropenia, they are managed empirically for bacteremia. However, a viral etiology is common and, in many cases, no definitive source of infection is identified, the fever and neutropenia resolve, and patients have a relatively short duration of hospitalization with favorable outcome.^1^, ^2^, ^3^, ^4^, ^5^ In March 2020, The United States declared a national emergency secondary to COVID‐19 and issued a stay‐at‐home order. The American Cancer Society subsequently released a statement encouraging individuals to postpone cancer screenings.^6^ Routine pediatric visits were postponed or canceled, and emergency rooms were reserved for the sickest patients requiring emergent evaluation.^7^ In August of 2021, a Global Registry of COVID‐19 in Childhood Cancer cohort showed that one‐fifth of children with cancer infected with COVID‐19 worldwide had severe illness, with an associated mortality rate exceeding that reported in the general pediatric population. Therefore, changes in cancer‐directed therapy, including delaying or withholding chemotherapy, occurred in significant numbers of pediatric cancer patients.^8^ On November 26, 2021 the World Health Organization designated Omicron (B.1.1.529) as a variant of concern which rapidly replaced Delta as the dominant variant globally soon after.^9^ The Omicron variant is more transmissible than the Delta Variant, but it is well documented that the risk of hospitalization and death is lower for Omicron cases compared to prior variants worldwide.^10^, ^11^

The Children's Hospital at Montefiore, an urban tertiary care academic hospital with a designated oncology floor, is the only children's hospital in the Bronx, the borough with the highest SARS‐CoV‐2 infection rate in New York City.^12^ We aimed to identify an association between COVID‐19 positivity and bacteremia, sepsis, or serious respiratory illness. We hypothesized that COVID‐19 positivity does not lead to bacteremia, sepsis, or serious respiratory illness in pediatric oncology patients. Sepsis was defined as life‐threatening organ dysfunction due to a dysregulated host response to infection. Therefore, we conducted a retrospective chart review of pediatric patients with cancer who tested positive for COVID‐19 from December 2021 until February 2022, during the Omicron surge. Although, viral genotyping was not completed, more than 90% of viral isolates beginning December 19, 2021, and 95%–100% beginning in January, were Omicron variants in both the Montefiore Health System and NYC.^13^

## 2 METHODS

We conducted a retrospective analysis of all pediatric hematology/oncology patients presenting to a single center site who tested positive for COVID‐19 between December 20, 2021 and February 22, 2022. In order to be included in our cohort, patients must have followed with our Oncology team in clinic or had a bone marrow transplant followed by our service. Additionally, they must have tested positive for COVID‐19 as indicated by an in‐hospital polymerase chain reaction COVID nasal swab. Cases were identified through weekly pediatric oncology sign outs between faculty. Our cohort was comprised of 26 patients (20 male and 6 female), between 2 and 24 years of age. We collected each patient's date of COVID‐19 positivity, COVID‐19 treatment received, COVID‐19 vaccination status, phase of cancer therapy at time of COVID‐19 diagnosis, and history of bacteremia. Chart review was conducted for each patient to assess for previous history of bacteremia. Bacteremia was described as a positive blood culture necessitating antibiotic therapy. The included patients are representative of the diversity of diagnoses we see in our urban tertiary care academic children's hospital pediatric oncology practice.

## 3 RESULTS

Of the 26 patients, sixteen had solid tumors, lymphoma, or leukemia (Table 1). Twelve patients were unvaccinated (U) at the time of COVID‐19 diagnosis, four were fully vaccinated (F), and zero patients received the booster (B) (Figure S1). Of the fully vaccinated individuals, 75% were eligible for the COVID‐19 booster at time of COVID‐19 diagnosis. Two patients with leukemia tested positive for COVID‐19 during induction, four during consolidation, and four during continuation/maintenance. Three patients had a history of bacteremia. Seven of the 16 patients with solid tumors, lymphoma, or leukemia who tested positive for COVID‐19 received antiviral therapy that included a combination of Remdesevir, Paxlovid, or Sotrovimab, while nine patients did not receive any therapy. All patients from this cohort were discussed with our infectious disease specialists and qualified for antiviral treatment based on their immunocompromised state. 50% of patients aged 0–12 years old received antiviral therapy, 17% of patients aged 12–18 received antiviral therapy, and 100% of patients aged above 18 years old received antiviral therapy.

**TABLE 1:** Breakdown of cancer type, vaccination status, management, and outcome for patients diagnosed with COVID‐19

	Total (male/female)	Age range (years)	History of bacteremia	Vaccination status (U/P/F/B)	No treatment	Antiviral therapy	Steroids	COVID‐19 associated respiratory support	COVID‐19 associated sepsis
Leukemia/lymphoma	10 (7/3)	2–22	3	8/0/2/0	5	5	0	0	0
Solid tumors	3 (2/1)	12–22	0	2/0/1/0	2	1	0	0	0
CNS tumor	1 (1/0)	13	0	1/0/0/0	1	0	0	0	0
Other malignancy	2 (2/0)	12–18	0	1/01/0	1	1	0	0	0

Ten patients who previously underwent hematopoietic stem cell transplant (HSCT) tested positive for COVID‐19 during this timeframe (Table 2). Six were less than 1‐year post‐stem cell transplant and four were greater than 1‐year post transplant. Three patients who were within 1 year of HSCT from date of COVID‐19 positivity had a history of prior bacteremia, while two patients who had a HSCT greater than 1 year prior to COVID‐19 positivity had a history of bacteremia. Of the 10 HSCT patients who tested positive for COVID‐19, 8 did not receive a COVID‐19 vaccination prior to diagnosis (U), 2 were fully vaccinated (F), and 0 patients received a booster (B) (Figure S2). Of the fully vaccinated individuals, 2 were eligible for the booster at time of COVID‐19 diagnosis. One patient with a HSCT within 1‐year did not receive treatment, while five received antiviral therapy. Three patients greater than 1‐year post HSCT did not receive COVID‐19 treatment, and one received antiviral therapy and steroids.

**TABLE 2:** Breakdown of hematopoietic stem cell transplant timeframe, vaccination status, management, and outcome for patients diagnosed with COVID‐19

	Total (Male/female)	Age range (years)	History of bacteremia	Vaccination status (U/P/F/B)	No treatment	Antiviral therapy	Steroids	COVID‐19 associated respiratory support	COVID‐19 associated sepsis
<1‐year post stem cell transplant	6 (4/2)	2–19	3	6/0/0/0	1	5	0	0	0
>1‐year post stem cell transplant	4 (4/0)	8–24	2	2/0/0/0	3	1	1	0	0

*Note*: U/P/F/B = unvaccinated/partially vaccinated/fully vaccinated/boosted.

No patients were admitted for respiratory support or bacteremia after a diagnosis of COVID‐19, with respiratory support defined as nasal cannula or high‐flow oxygen delivery, positive pressure ventilation, or intubation. Routine labs, including complete blood counts, would have been obtained to assess for neutropenia if patients were febrile or required hospital admission. During illness and after a COVID positive result, when patients were contacted to follow up on symptoms, no patients required hospital admission, which is indicative of mild infection not associated with fever/neutropenia or vomiting/diarrhea/dehydration. This data shows that among our cohort, no patients became significantly ill or developed concurrent bacteremia due to COVID‐19 during the Omicron surge.

## 4 DISCUSSION

We studied if COVID‐19 infection during the Omicron surge was associated with bacteremia or serious respiratory illness in our patient population. Pediatric cancer patients receiving chemotherapy are vulnerable to infection, though we found that none of the patients infected with the Omicron variant of COVID‐19 developed bacteremia or became severely ill. The strength of this study is that our patients are representative of the greater pediatric oncology community with diversity of diagnoses, phase of treatment at the time of COVID‐19 diagnosis, and history of bacteremia. However, it is a relatively small observational study limited by confounding variables and potential bias, and a casual relation cannot be definitely established. It is also not known if intrinsic host or Omicron‐specific viral properties led to less severe disease in our patient population; at this point in the pandemic, it would be expected that patients have defenses from previous infections and/or vaccine‐induced immunity.^14^

Oncologists often recommend a variety of infection control measures, like regular handwashing and avoiding known sick contacts to avoid febrile neutropenia given that 10%–20% of cases are associated with bacteremia or serious infection and the mortality risk is 0.7%–3.9%.^1^, ^15^, ^16^, ^17^, ^18^ However, guidance varies considerably among medical providers about which activities, such as attending school, utilizing public transportation, frequenting crowded areas, and playing on playgrounds, that afebrile neutropenic children with cancer can safely engage in.^19^, ^20^ Underlying the variable infection control recommendations is the false conflation of community‐acquired infection and serious bacterial infection in febrile neutropenic patients. Intrinsic properties, such as the loss of gastrointestinal tract integrity and the presence of central venous catheters, as opposed to community inquired infections like COVID‐19, are typically implicated in bacteremia.

COVID variants are likely going to continue to affect treatment plans of pediatric patients undergoing cancer‐directed therapy, and our findings suggest no association between COVID‐19 infection and life‐threatening bacteremia. Larger studies will improve our understanding about extrinsic infectious disease risk factors and intrinsic bacteremia risk so the guidance we give our patients can become standardized and reflect the understanding that community‐acquired infections do not drive bacteremia in pediatric oncology patients. This is critically important to guide clinical decision‐making and allow pediatric oncology patients to live as fulfilling of a life as possible, unhindered by overly cautious recommendations.

## AUTHOR CONTRIBUTIONS

**Rachel Offenbacher:** Conceptualization (lead); data curation (lead); formal analysis (lead); methodology (equal). **Joshua Gorlick:** Investigation (equal); methodology (equal); writing – original draft (equal); writing – review and editing (equal). **Daniel A Weiser:** Conceptualization (equal); investigation (equal); methodology (equal); project administration (equal); writing – original draft (equal); writing – review and editing (equal).

## CONFLICT OF INTEREST

The authors declare no conflict of interest.

## ETHICS STATEMENT

This study was approved through the Einstein IRB; 2019‐10232.

## Supporting information


**Figure S1:** Breakdown of Age, Vaccination Status, Cancer Type, Management, and Outcome for Patients Diagnosed with COVID‐19
**Figure S2:** Breakdown of Age, Hematopoietic Stem Cell Transplant Timeframe, Vaccination Status, Management, and Outcome for Patients Diagnosed with COVID‐19

## Data Availability

N/A.
