# Supplementary material for: Omicron variant of SARS‐CoV‐2, an epidemiologic assessment of pediatric oncology patients in the Bronx
Source: Cancer Rep (Hoboken). 2022 Oct 5;5(11):e1724. doi: 10.1002/cnr2.1724 (PMC9675379; doi:10.1002/cnr2.1724)
Supplement: Supplementary file 1 — Figure S1: Breakdown of Age, Vaccination Status, Cancer Type, Management, and Outcome for Patients Diagnosed with COVID‐19 Figure S2: Breakdown of Age, Hematopoietic Stem Cell Transplant Timeframe, Vaccination Status, Management, and Outcome for Patients Diagnosed with COVID‐19 [file CNR2-5-e1724-s001.docx]

Supplementary Figure 1: Breakdown of Age, Vaccination Status, Cancer Type, Management, and Outcome for Patients Diagnosed with COVID-19

| Age Range  (years) | Total (Male/Female) | Cancer Type  (S/L/C/O) | History of Bacteremia | Vaccination Status (U/P/F/B) | No Treatment | Antiviral Therapy | Steroids | COVID-19 associated respiratory support | COVID-19 associated sepsis |
| --- | --- | --- | --- | --- | --- | --- | --- | --- | --- |
| 0-5 | 4 (3/1) | 0/4/0/0 | 1 | (4/0/0/0) | 2 | 2 | 0 | 0 | 0 |
| 5-12 | 2 (1/1) | 0/2/0/0 | 0 | (2/0/0/0) | 1 | 1 | 0 | 0 | 0 |
| 12-18 | 7 (5/2) | 2/3/1/1 | 2 | (5/0/2/0) | 6 | 1 | 0 | 0 | 0 |
| >18 | 3 (3/0) | 1/1/0/1 | 0 | (1/0/2/0) | 0 | 3 | 0 | 0 | 0 |

Supplementary Figure 2: Breakdown of Age, Hematopoietic Stem Cell Transplant Timeframe, Vaccination Status, Management, and Outcome for Patients Diagnosed with COVID-19

| Age Range  (years) | Total (Male/Female) | Time post stem cell transplant  (<1, >1 yr) | History of Bacteremia | Vaccination Status (U/P/F/B) | No Treatment | Antiviral Therapy | Steroids | COVID-19 associated respiratory support | COVID-19 associated sepsis |
| --- | --- | --- | --- | --- | --- | --- | --- | --- | --- |
| 0-5 | 1 (1/0) | (1/0) | 0 | (1/0/0/0) | 1 | 0 | 0 | 0 | 0 |
| 5-18 | 6 (5/1) | (4/2) | 4 | (5/0/1/0) | 2 | 4 | 0 | 0 | 0 |
| >18 | 3 (2/1) | (1/2) | 1 | (1/0/2/0) | 1 | 2 | 1 | 0 | 0 |

****S/L/C/O=solid tumor/leukemia+lymphoma/CNS tumor/other malignancy
